# A chromatin modifying enzyme, SDG8, is involved in morphological, gene expression, and epigenetic responses to mechanical stimulation

**DOI:** 10.3389/fpls.2014.00533

**Published:** 2014-10-21

**Authors:** Christopher I. Cazzonelli, Nazia Nisar, Andrea C. Roberts, Kevin D. Murray, Justin O. Borevitz, Barry J. Pogson

**Affiliations:** ^1^Hawkesbury Institute for the Environment, University of Western SydneyPenrith, NSW, Australia; ^2^Australian Research Council Centre of Excellence in Plant Energy Biology, Research School of Biology, College of Medicine, Biology and Environment, The Australian National UniversityCanberra, ACT, Australia

**Keywords:** acclimation, Arabidopsis, histone, touch, transcription, thigmomorphogenesis, mechanical, regulation

## Abstract

Thigmomorphogenesis is viewed as being a response process of acclimation to short repetitive bursts of mechanical stimulation or touch. The underlying molecular mechanisms that coordinate changes in how touch signals lead to long-term morphological changes are enigmatic. Touch responsive gene expression is rapid and transient, and no transcription factor or DNA regulatory motif has been reported that could confer a genome wide mechanical stimulus. We report here on a chromatin modifying enzyme, SDG8/ASHH2, which can regulate the expression of many touch responsive genes identified in Arabidopsis. SDG8 is required for the permissive expression of touch induced genes; and the loss of function of *sdg8* perturbs the maximum levels of induction on selected touch gene targets. SDG8 is required to maintain permissive H3K4 trimethylation marks surrounding the Arabidopsis touch-inducible gene TOUCH 3 (TCH3), which encodes a calmodulin-like protein (CML12). The gene neighboring was also slightly down regulated, revealing a new target for SDG8 mediated chromatin modification. Finally, *sdg8* mutants show perturbed morphological response to wind-agitated mechanical stimuli, implicating an epigenetic memory-forming process in the acclimation response of thigmomorphogenesis.

## Introduction

Mechanical forces imposed by environment stimuli such as touch, strong winds, rubbing, tree strangling, insect feeding, passing animals, weight of climbing plants, heavy rain and even the navigation of roots around obstacles in the soil, can be perceived by plants (Braam, 2005; Monshausen and Haswell, 2013). When plants are grown in the wind-protected whole tree chambers, greenhouses, or growth chambers, it is possible to separate out the long term effects of mechanical stimulation from other natural environmental and seasonal climatic transitions that shape plant development. Repeated touching of plant organs for short periods of time eventually alters growth leading to phenotypic changes, which is a phenomenon referred to as thigmomorphogenesis (Jaffe, 1973; Chehab et al., 2009). The sensitivity of plants to repeated mechanical stress alters the degree of carbon allocation and the shoot-root biomass balance. Plants physiologically and morphologically acclimate to these mechanical wind forces that threaten reproduction and survival (Biddington, 1986; Mitchell, 1996; Coutand et al., 2008).

Although thigmomorphogenesis is perceived as a slow response to mechanical perturbation, there are very fast physiological changes associated with mechanical stress. These rapid responses can affect photosynthesis and respiration by impacting the resistance to movement of carbon dioxide into leaves and altering stomatal aperture (Jaffe and Forbes, 1993; Smith and Ennos, 2003). Long term morphological changes can include internode compression, pithiness, decreased rate of stem and petiole elongation, lateral or radial enlargement (i.e., swelling) of stems, inhibition of leaf expansion. Developmental changes include enhanced senescence, delayed flowering, and stronger roots (Jaffe and Forbes, 1993; Braam, 2005; Chehab et al., 2009). Other responses include enhanced pest resistance and decreased susceptibility to various stresses as well as alterations in chlorophyll content and hormone levels (Biddington, 1986; Tretner et al., 2008; Chehab et al., 2012; Monshausen and Haswell, 2013).

Various signaling pathways and molecules function interdependently to mediate mechanical stimulation of physiological and morphological responses to touch. Characteristic early signaling events of touch-induced responses involve secondary messenger molecules (calcium, nitric oxide and reactive oxygen species) and protein phosphorylation (Braam and Davis, 1990; Hofmann, 2009; Monshausen et al., 2009; Kurusu et al., 2012a,b). Touch-induced genes identified encode calmodulin (TCH1), calmodulin-like-proteins (TCH2 and TCH3) and a xyloglucan endotransglucosylase/ hydrolase (TCH4), all known to play a central role in touch signal transduction (Chehab et al., 2009). In addition, more recent progress has identified various putative mechanoreceptors including mechano-sensitive ion channel-like proteins and receptor-like kinases, which has paved further exploration toward understanding mechanical signal transduction (Kurusu et al., 2013; Monshausen and Haswell, 2013). Later signaling events involve hormones (jasmonates, ethylene, abscisic acid, auxin, brassinosteroids) that can confer the external environmental stimulus to the nucleus and promote morphological adaptation (Chehab et al., 2009). The phytohomones auxin and jasmonic acid (JA), play key roles in promoting thigmomorphogenesis. In particular, jasmonates have been shown to be required for, and promote, the salient characteristics of thigmomorphogenesis in *Arabidopsis*, including a touch induced delay in flowering and rosette diameter reduction (Chehab et al., 2012).

The physiological and morphological changes resulting from mechanical stimulation require alterations in gene expression and the production of new proteins. *TCH* gene expression can be observed at sites of potential mechanical strain and/or increased growth, such as the shoot branching points, root–shoot junction, elongating hypocotyls and roots, as well as developing trichomes (Sistrunk et al., 1994; Antosiewicz et al., 1995; Xu et al., 1995). A genome-wide differential expression analysis of mechanical stimulated leaves revealed that over 2.5% of *Arabidopsis* genes were up-regulated by at least 2-fold in response to touch stimulation (Lee et al., 2005). The vast majority of *TCH* regulated genes in Arabidopsis encode protein kinases, transcription factors and putative disease resistance proteins. They function in various cellular processes including calcium sensing/binding, cell wall modifications, and defense (Lee et al., 2005). Consistent with these findings, the molecular basis of the touch response has been implicated with multiple biotic and abiotic stimuli, including hormones, darkness, salt, and temperature (Braam, 2005). Large upstream promoter regions from the TCH4/XTH22 (xyloglucan endotransglucosylase/ hydrolase), CBF2/DREB1c (ERF/AP2 transcription factor), and TCH2/CML24 (Calmodulin-like/calcium binding protein) genes have been shown to be touch responsive (Iliev et al., 2002; Zarka et al., 2003; Braam, 2005). A transcriptional regulator known as Jr-ZFP2, encodes a Cys2/His2-type two-zinc-fingered protein and mRNA expression was shown to be associated with an acclimation response to mechanical bending (Leblanc-Fournier et al., 2008; Coutand et al., 2009; Martin et al., 2009). However, despite all these findings, not a single well characterized touch-inducible *cis*-acting element has been identified to date. Perhaps a more complex regulatory layer is involved whereby multiple signals converge to stimulate touch induced expression of select gene targets.

Coordinating a large number of TCH-inducible gene changes may require a degree of chromatin modification. Touch gene expression needs to be precisely timed and coordinated during all developmental stages as well as in the response to other environmental changes. Plant tissues that are mechanically perturbed by wind, rain or touch show a rapid and transient change in gene expression, usually within 5–30 min, that in some cases can be undetectable at basal levels in untouched tissues (Botella et al., 1992; Cazzonelli et al., 2005; Chehab et al., 2009). One of the central regulators of gene transcription is conferred through the organization of the genome into chromatin (Cazzonelli et al., 2009b). Histone proteins are key components of chromatin, forming the basic nucleosome packaging structure. Posttranslational modifications, such as the methylation of lysine residues on the tails of histone proteins can activate (e.g., H3K4, H3K36) or repress (e.g., H3K9, H3K9) gene expression depending upon changes in the environment (e.g., drought, cold and high-salinity stress) or developmental cues (Cazzonelli et al., 2009b; Kim et al., 2010; Berr et al., 2011b; Song et al., 2013). Histone modifications can be read, written, and edited, which highlights their plasticity to tune regulatory processes (Justin et al., 2010). In particular, histone lysine methylation (HKM) can promote strong and inducible target gene expression within a very short period of time of receiving a stress stimulus (Lim et al., 2009; Kim et al., 2010). Such characteristics of an epigenetic mark could be envisioned as important for conferring a nuclear wide response to a mechanical stimulus.

In order to address the molecular nature behind the phenomenon of thigmomorphogenesis, we have investigated if chromatin regulatory mechanisms. This includes the well-studied permissive modification of trimethylation of H3K4, which can enhance the expression of mechanical-induced genes. We provide evidence to show that many gene targets regulated by the chromatin modifying gene, SET DOMAIN GROUP 8 (SDG8) are also responsive to mechanical stimulation in *Arabidopsis* (Cazzonelli et al., 2009a, 2010). The loss of function of *sdg8* altered morphological responses to long-term mechanical stimulation revealing a potential new layer in the regulation of thigmormorphogenesis, and provides a basis for further understanding the molecular regulation of this enigmatic phenomenon.

## Methods and materials

### Plant growth conditions and germplasm

Soil grown plants were incubated at 4°C for 2–3 days in the dark before transferring to 12 h of illumination (100–150 μE) and temperature maintained at 21°C. All germplasm are in the Arabidopsis thaliana ecotype Columbia (Col-0) background and mutants used in this study include *chloroplast carotenoid regulation 1-1* (*ccr1-1*; referred to as *sdg8-1*) and *ccr1.4* (referred to as *sdg8-4*), which have a null lesion in SDG8/ASHH2, a histone lysine methyltransferase; AT1G77300 (Cazzonelli et al., 2009a).

### Mechanical stimulation of TCH gene expression and thigmomorphogenesis

A mechanical force was applied by lightly bending mature Arabidopsis (29 DAG) leaves 30 times for 30 s with four repeats and harvesting tissues 30 min following stimulation. To promote thigmomorphogenesis, a treatment of constant mechanical force was applied to unstimulated plants (20 DAG; 6–13 true leaves; no sign of floral bolt) growing in separate trays using stationary fans (twice a day for 15 min). The stimulation was visible as leaf vibration. Three additional trays of control plants were shielded from the mechanical stimulation using cardboard boxing. A constant temperature of 21°C was maintained for both the wind-stimulated and control plants. After 7 days fan-forced mechanical stimulation was stopped and photos taken 2 days later for further analysis of morphological traits (e.g., leaf blade length and width, and petiole length). Experimental set up involved growing 8–15 plants for each wild type and *sdg8* mutant in separate trays. Plant level measures were taken as the average of 5 fully expanded rosette leaves calibrated to the standard pot size using image processing software ImageJ2 (Schneider et al., 2012).

### Real time quantitative PCR

Total RNA was extracted using the SIGMA-aldrich Spectrum kit and included an on-column DNase treatment step following the manufacturer's instructions. First strand cDNA synthesis was performed using Oligo dT primer and SuperScript® III Reverse Transcriptase (Invitrogen) according to manufacturer's instructions. The relative transcript abundance was quantified using Light Cycler 480 SYBR Green I Master and three technical replicates for each of one to three biological replicates were performed using the Light Cycler 480 (ROCHE, Australia). The take-off point was determined using relative quantification [Target Eff Ct(Wt-target) / Reference Eff Ct(Wt-target)] and fit point analysis (Pfaffl, 2001). *Cyclophilin* (At2g29960) and *Protein Phosphatase 2A* (At1g13320) were included as house-keeper reference control genes (Czechowski et al., 2005). Primer sequences are listed in Supplemental Table 1.

### Bioinformatics analysis of microarray data and gene ontology anlysis

Analysis of *sdg8* microarray data was obtained from the following sources: mature leaves (Cazzonelli et al., 2009a), 6 DAG seedling leaves (Xu et al., 2008) and inflorescences (Grini et al., 2009), as well as wild type touch induced leaves (Lee et al., 2005). Gene ontology analysis was performed using the agriGO -GO Analysis Toolkit and Database for Agricultural Community (Du et al., 2010).

### Chromatin immunoprecipitation

Chromatin immunoprecipitation (ChIP) assays were performed using a pool of 3-week-old leaf tissue as previously described (Cazzonelli et al., 2009a). There were three biological replicates for each of the two genotypes analyzed. The chromatin/DNA extracted from leaf pools was divided into three aliquots, two of which were used for different antibodies and the third used as a control to which no antibody was added. Antibodies recognizing H3K4me3 (Millipore Cat#04-745) and H3K4me2 (Millipore Cat#07-030) were purchased from Upstate Biotechnology (Charlottesville, VI, USA). The no antibody control was included to verify that H3K4 antibodies were able to enrich ChIP DNA by at least 10-fold. ChIP DNA was tested in triplicate by PCR for enriched regions of DNA and normalized to the housekeeping gene S-Adenosyl Methionine Synthase, (SAM; At4g01850) (Finnegan et al., 2004). qRT-PCR primers used for ChIP analysis are given in Supplemental Table 2.

### Data analysis

**Figure 1:**
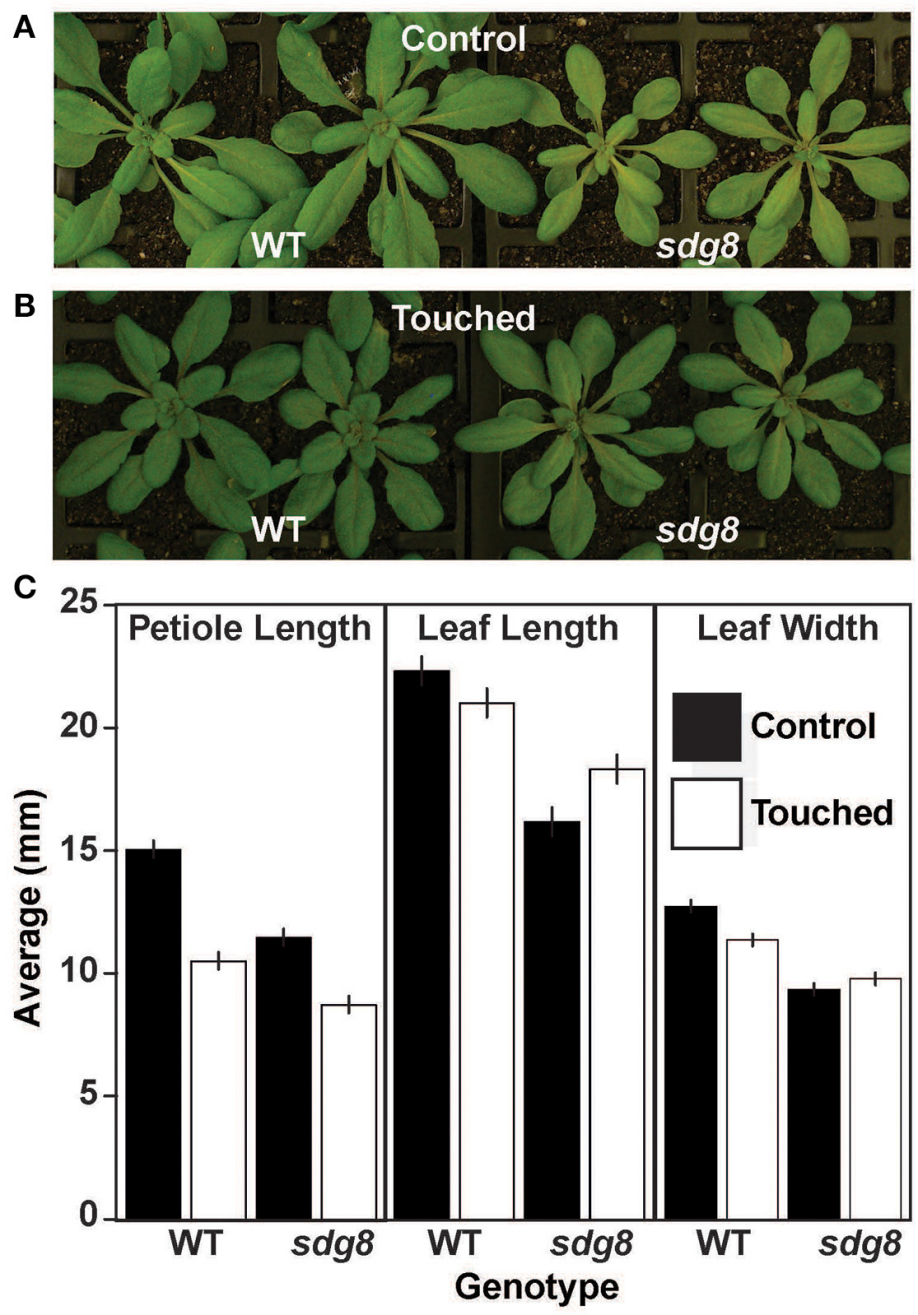
**Thigmomorphogenic responses of the *sdg8* mutant to mechanical stress**. Wild type (WT) and *sdg8* mutant Arabidopsis plants were grown under controlled conditions **(A)** or subject to 1 week of short, repetitive bursts of wind-agitated mechanical stimulation **(B)**. **(C)** Morphological traits (petiole length, leaf blade length and leaf blade width) were measured for WT and *sdg8*, with and without mechanical treatment. *N* = 449 plants. Error bars are displayed (+/− twice the standard error).

Data analyses were performed in R using the nlme package. 449 plants from six trays each with WT and *sdg8* were analyzed. Three trays were treated with fans to provide mechanical stress from wind. A mixed effect model was used to estimate the fixed effects of genotype, treatment, and genotype by treatment interaction and the random effect of Tray, as lme(TraitValue ~ geno^*^Treatment, random = ~1|Tray). The fitted values from each of the genotype and treatment classes are shown in Figure 1C.

## Accession numbers

Arabidopsis Genome Initiative locus identifiers for the genes mentioned in this article are as follows: At1g77300 (*SDG8; SET DOMAIN GROUP 8*), At1g06810 (*CYCLO*; *CYCLOPHILIN*), At1g13320 (*PP2A*; *PROTEIN PHOSPHATASE 2A*), AT4g34270 (TIP41; TIP41-LIKE FAMILY PROTEIN), At4g01850 (*SAM; S-ADENOSYL METHIONINE TRANSFERASE*), AT2G41100 (*TCH3; CALMODULIN-LIKE PROTEIN*), AT2G47060 (*STPK*; *SERINE/THREONINE PROTEIN KINASE*); AT4G23810 (*WRKY; DNA-BINDING PROTEIN 53*), At1g06820 (*CRTISO; CAROTENOID ISOMERASE*).

## Results

### Microarray analysis reveals SDG8 regulates touch responsive gene expression

Active chromatin modifications are required for inducible gene expression. SDG8 is a well characterized chromatin-modifying enzyme that catalyzes the trimethylation of active marks of histone-3-lysine-4 (H3K4) and histone-3-lysine-36 (H3K36) at selected permissive gene loci involved in carotenogenesis, flowering, and defense responses (Kim et al., 2005; Cazzonelli et al., 2009a; Berr et al., 2010). Previously published microarray analyses of mature leaf tissues from the *sdg8* mutant revealed mis-regulation of a mechanical responsive *TOUCH3* (*TCH3*) gene, which encodes a calmodulin-like protein (CML12) (Sistrunk et al., 1994; Antosiewicz et al., 1995) (Supplemental Table 1) (Cazzonelli et al., 2009a). The genes neighboring *TCH3* were also slightly down-regulated (*p* < 0.05; data not shown) and consistent with previous reports demonstrating that active chromatin marks can spread to surrounding gene loci (Cazzonelli et al., 2009a). These findings paved the way for a more thorough bioinformatics analysis to determine if SDG8 plays a more prominent role in the regulation of touch induced gene expression.

We next analyzed four separate genome-wide transcript studies of different tissues (mature leaves, whole seedlings and inflorescences) from different *sdg8* mutant alleles (Supplemental Table 1). These studies identified the majority of differentially expressed genes to be down-regulated (65–75%; Table 1) and consistent with the function of SDG8 in actively promoting gene expression (Cazzonelli et al., 2009a). Further analysis revealed many differentially expressed genes in *sdg8* were also touch responsive in leaves (8.7%), seedlings (16–40%) and to some small extent inflorescences (3.3%; Table 1). The genes that were down-regulated in *sdg8* were largely inducible by mechanical stimulation (50–85%) and consistent with the function of SDG8 in promoting a more permissive chromatin structure that activates gene expression (Table 1).

**Table 1:** **Differentially expressed genes regulated by SDG8 and mechanical stimulation**.

**Microarray publication**	**Cazzonelli et al., 2009a**	**Xu et al., 2008**		**Grini et al., 2009**	**Lee et al., 2005**
Genotype and/or treatment	*ccr1.1*	*sdg8-1*	*sdg8-2*	*ashh2-1*	Touch (TCH)
Tissue	Leaves	Seedlings	Seedlings	Inflorescence	Leaves
Differentially expressed genes	113	83	142	448	760
Genes down-regulated	85 (75%)	123 (67%)	93 (65%)	298 (67%)	171 (22%)
Genes up-regulated	28 (25%)	40 (33%)	49 (35%)	150 (33%)	589 (78%)
*TCH* responsive genes in *sdg8*	13 (8.7%)	33 (40%)	22 (16%)	15 (3.3%)	65 (8.6%)
*TCH* inducible and down-regulated in *sdg8*	11 (85%)	29 (88%)	11 (50%)	0	57 (88%)

Comparison of touch inducible genes regulated by *sdg8* identified 65 differentially co-expressed genes (8.6%) of which the majority were down-regulated in the *sdg8* mutant and touch inducible (>88%; Table 1 and Supplemental Table 1). Gene ontology analysis of the 65 differentially expressed genes showed significant (FDR < 0.00005) enrichment in transcripts involved in response to abiotic stress (cold temperature and oxidative damage), defense (chitin, fungus, and immune system), chemicals (carbohydrates and organic substances), wounding and other external stimuli (Table 2). These gene classes have often been implicated in the activation of touch responsive genes (Braam, 2005). Furthermore, these results mirrored the gene ontology analysis of 760 touch responsive genes identified in Arabidopsis (Supplemental Table 1). In summary, there were many genes down-regulated in the *sdg8* mutant that were also inducible by mechanical stimulation.

**Table 2:** **Gene ontology analysis of touch inducible genes regulated by mechanical stimulation in the sdg8 mutant**.

**GO term**	**Ontology process**	**Description**	**Co-reg. Genes**	**Ref. genes**	**FDR**
GO:0010200	P	Response to chitin	11	151	1.30E-12
GO:0050896	P	Response to stimulus	31	4057	1.10E-11
GO:0009743	P	Response to carbohydrate stimulus	11	240	5.30E-11
GO:0042221	P	Response to chemical stimulus	22	2085	1.90E-10
GO:0006950	P	Response to stress	22	2320	1.20E-09
GO:0010033	P	Response to organic substance	17	1342	4.20E-09
GO:0006952	P	Defense response	13	766	2.60E-08
GO:0009605	P	Response to external stimulus	8	429	2.90E-05
GO:0009611	P	Response to wounding	6	197	4.30E-05
GO:0006955	P	Immune response	7	367	9.40E-05
GO:0002376	P	Immune system process	7	368	9.40E-05
GO:0051704	P	Multi-organism process	9	776	1.80E-04
GO:0051707	P	Response to other organism	8	599	1.90E-04
GO:0009620	P	Response to fungus	5	158	1.90E-04
GO:0009607	P	Response to biotic stimulus	8	638	2.70E-04
GO:0009266	P	Response to temperature stimulus	6	485	3.30E-03
GO:0009628	P	Response to abiotic stimulus	10	1471	3.30E-03
GO:0009409	P	Response to cold	5	328	4.20E-03
GO:0006979	P	Response to oxidative stress	5	332	4.20E-03
GO:0045087	P	Innate immune response	5	347	4.90E-03
GO:0050794	P	Regulation of cellular process	14	3375	2.10E-02
GO:0009416	P	Response to light stimulus	5	596	4.50E-02
GO:0050789	P	Regulation of biological process	14	3697	4.50E-02
GO:0009314	P	Response to radiation	5	613	4.70E-02
GO:0010556	P	Regulation of macromolecule biosynthetic process	9	1843	4.70E-02
GO:0031326	P	Regulation of cellular biosynthetic process	9	1881	5.00E-02
GO:0009889	P	Regulation of biosynthetic process	9	1881	5.00E-02

*The agriGO – gene ontology analysis toolkit was used to determine responses in 65 genes regulated by SDG8 and mechanical stimulation*.

### Thigmomorphogenic responses are perturbed in the *sdg8* mutant

Mechanical stimulation of Arabidopsis plants by wind-forced agitation promoted a large thigmomorphogenic response in wild type (Figures 1A,B) that was consistent with previous reports (Braam and Davis, 1990; Braam, 2005). The morphological traits altered included a shortening of petiole length and a reduction in leaf blade length and width (Figure 1C). In contrast, the *sdg8* mutant had perturbed responses to stimulation (*p* < 0.01 differential sensitivity for all traits) displaying a minor reduction in petiole length, an increase in leaf blade length and no response in leaf width. Overall, the *sdg8* mutants were less sensitive to mechanical stress (Data Analysis S1 in Supplementary Material).

### TCH gene expression levels are reduced in *sdg8* mutant tissues

Next we decided to validate the relative transcript levels of three touch inducible genes (*WRKY53, STPK* and *TCH3*), which were identified as significantly down-regulated in *sdg8* and inducible by mechanical stimulation (*ccr1.1*; Supplemental Table 1). These genes were chosen for the following reasons; (1) *WRKY53* (DNA-binding protein transcription factor) showed the highest level of touch induction (>11 fold) with a 2.5 fold down-regulation of expression in *sdg8*, (2) *STPK* (putative serine/threonine protein kinase) was down-regulated in three independent microarray studies (2–4 fold) and up-regulated 4.2 fold by touch simulation, and (3) *TCH3* (calcium signaling protein whose gene expression has been well characterized as touch inducible) was down regulated 3.7 fold in *sdg8* and up-regulated by 2.4 fold following mechanical stimulation.

**Figure 2:**
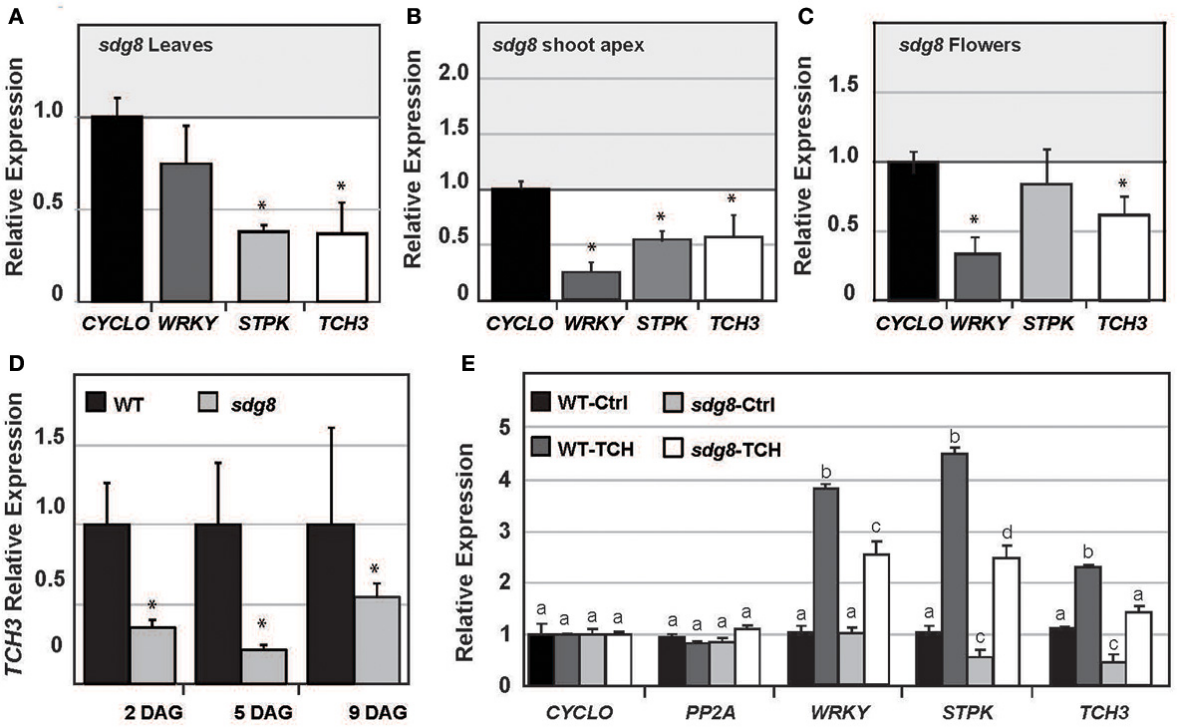
**Relative transcript levels of touch-responsive genes in *sdg8* mutant tissues**. Leaf and floral tissues were pooled from independent plants and qRT-PCR used to quantify gene expression levels from three biological replicates in mutant *sdg8* lines and normalized to wild type. **(A–C)** Relative gene expression levels of *WRKY53, STPK* and *TCH3* in *sdg8* mutant rosette leaves (21 DAG), shoot apical meristem (21 DAG) or floral tissues (35 DAG), respectively. **(D)** *TCH3* transcript levels in wild type and *sdg8* mutant whole seedlings tissues. **(E)** Relative expression levels of *WRKY53, STPK* and *TCH3* in mechanically stimulated mature rosette leaves. Values above the bar sets represent an ANOVA *p*-value (<0.05 signifies more than 95% confidence) considering the interaction of genotype by touch treatment. Relative expression levels represent ratios normalized to wild type and using house keeper reference genes (*CYCLO, PP2A*, or *TIP41*). Standard error bars represent 2–3 biological repeats and up to 2 experimental repeats (*n* = 2–6). ^*^Denotes *p* < 0.05 (unpaired Welch's *T*-Test). Primer sequences are given in Supplemental Table 2. Unless otherwise specified, plants were grown in controlled environments without any mechanical stimulation.

The relative levels of *WRKY53, STPK* and *TCH3* mRNA expression were quantified in rosette leaves, shoot apices, and floral tissues of *sdg8* (Figures 2A–C). The transcript levels of *WRKY* and *STPK* were significantly reduced in the shoot apex, as well as flowers and leaves, respectively. *TCH3* mRNA levels were also significantly reduced (40–60%) in these *sdg8* mutant tissues as well as during seedling development (>50%) (Figure 2D).

Mechanical stimulation of leaf tissues by bending for 30 s (tissue harvested 30 min after touch stimulus) significantly enhanced the relative transcript levels of *WRKY53, STPK*, and *TCH3* in wild type (~2–5 fold above unstimulated control leaves), and to a lesser degree in the *sdg8* mutant (Figure 2E). The interaction between mutant and touch treatment was significantly compromised for *WRKY* (*p* < 0.01). Transcript levels in *sdg8* were similar to wild type and the induction of *WRKY* mRNA levels following touch stimulation was less in *sdg8* when compared to wild type (Figure 2E). The transcript levels of *STPK* and *TCH3* in *sdg8* was significantly lower in both untreated and touched leaf tissues when compared to wild type (Figure 2E), however the fold change in touch induction of *TCH3* and *STPK* gene expression in *sdg8* mutants (3- and 4-fold respectively) was similar to WT plants (2- and 4-fold, respectively). It should be noted that our experiments addressed a single time point of 30 min to keep consistent with previous TCH microarray data (Lee et al., 2005). In summary, SDG8 is required to promote high levels of *TCH* gene expression following mechanical stimulation, but does not appear to be necessary for activating the induction of *TCH* gene expression.

### Chromatin surrounding TCH3 gene locus shows reduced H3 lysine 4 trimethylation

**Figure 3:**
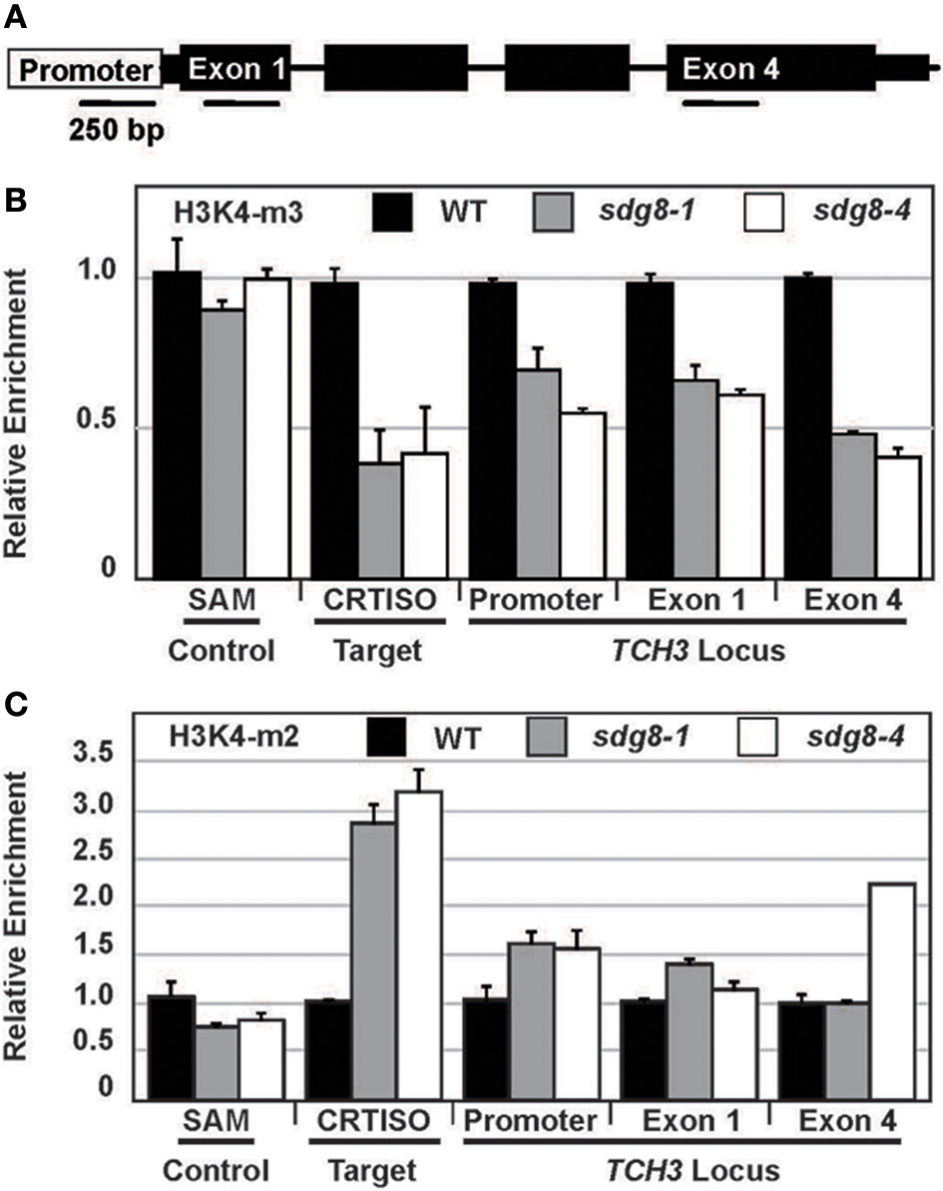
**Relative enrichment of histone 3 lysine 4 methylation of chromatin surrounding the *TCH3* locus in the *sdg8* mutants. (A)** The position of PCR amplicons used to quantify H3K4 methylation associated with the TCH3 locus is schematically represented. Primer sequences are presented in Supplemental Table 2 online. **(B,C)** The level of H3K4me3 **(B)** and H3K4me2 **(C)** surrounding TCH3 chromatin is presented as a ratio mutant/wild type, following normalization using a region of the house-keeping gene, S-ADENOSYL METHIONINE SYNTHASE (SAM). CRTISO was included as a positive control for H3K4 di- or tri-methylation activity. For most amplicons two biological and 3 technical repeats were performed, except for TCH3-exon2 where only a single biological repeat was successful. In all experiments there was an enrichment of at least 10 fold above the no antibody background.

Next we established if *TCH3* was a target of SDG8 mediated chromatin modification. Chromatin immunoprecipitation of DNA from Arabidopsis leaf tissues using antibodies against H3 trimethylK4 (H3K4me3) and histone H3 dimethylK4 (H3K4me2) was followed by quantification using real-time PCR. One upstream (promoter) and two downstream (exon 1 and exon 4) regions flanking the *TCH3* translation start site (Figure 3A) were used to monitor the effect of SDG8 mutation on permissive histone marks surrounding *TCH3*. Genomic regions flanking *SAM* and *CRTISO* were also quantified as housekeeper and positive controls of histone methylation, respectively (Finnegan et al., 2004; Cazzonelli et al., 2009a). Analysis of the no antibody control samples revealed over 64-fold enrichment (>6 qPCR cycles) of ChiP precipitated DNA enriched in H3K4me3 and H3K4me2 marks.

Chromatin immunoprecipitation assays using antibodies recognizing H3K4 di- and tri-methylation (H3K4m3 and H3K4m2, respectively) showed that SDG8 activity perturbed permissive chromatin marks surrounding the *TCH3* promoter and two exon loci (Figure 3). In both *sdg8* mutant alleles (*sdg8-1 and sdg8-4*), there was an approximate 30–60% reduction in H3K4 trimethylation (Figure 3B), accompanied by an expected subtle increase in H3K4 dimethylation (Figure 3C). A similar reduction in the H3K4m3 and higher increase in the H3K4m2 marks were observed surrounding the *CRTISO* gene locus, which is consistent with previous findings (Cazzonelli et al., 2009a). Collectively, these data show that chromatin surrounding different regions spanning the *TCH3* locus have altered H3K4 methylation in *sdg8* alleles relative to wild-type plants, consistent with the decrease in *TCH3* transcript abundance.

The distribution of H3K4 methylation marks (mono, di and tri) as well as the nucleosome density of *TCH3, WRKY* and *STPK* was assessed using the UCSC Genome Browser (http://epigenomics.mcdb.ucla.edu/) (Zhang et al., 2009). Experimental derivations of marks surrounding the SAM reference gene showed no enrichment of H3K4m2, while H3K4m3 was restricted to the first intron (Figure S1A). Chromatin surrounding *WRKY53* and *TCH3* showed an enrichment in H3K4m2 and H3K4m3 extended throughout the gene locus (Figures S1B–D). The *WRKY* and *STPK* gene loci also showed an enrichment of H3K4m2 at the 5′ and 3′ untranslated mRNA leader regions (Figure S1B,C). All three touch inducible genes showed relatively no H3K4m1, which operates as an epigenetic mark for repressed euchromatin (Figure S1) (Van Dijk et al., 2005). The abundance of H3k4m2/m3 active marks of methylation surrounding the three touch inducible genes are consistent with transcriptional initiation (Cazzonelli et al., 2009b) and in good agreement with our findings showing a reduction in H3K4m3 marks surrounding the *TCH3* loci in *sdg8* mutant leaves (Figure S1).

## Discussion

Despite a widespread understanding of the signaling events leading to thigmomorphogenesis, the molecular nature that underpins plant mechanostimulus-induced gene expression remains largely unknown and open for discovery. Plants have evolved a very elaborate and sophisticated mechanical responsive regulatory network and this is illustrated by the fact that over 2.5% of Arabidopsis transcripts are responsive to touch stimulation (Lee et al., 2005). Yet, it is intriguing that not a single investigation has identified a *cis*-acting DNA regulatory element that can transcriptionally transmit the external touch stimulus to such a large array of mechanical responsive genes. Indeed, the characterization of a few upstream promoters from mechanical inducible genes have identified touch-responsive sequence domains (e.g., TCH2/TCH4; Chehab et al., 2009) as well as aberrant promoters that become deregulated and strongly constitutive rather than inducible (e.g., VRACS-1; Cazzonelli et al., 2005; Wever et al., 2010). Perhaps there are multiple signaling pathways required to activate gene expression following touch stimulation, rather than just relying upon a single universal mechanism.

Our discovery that SDG8 regulates 8.7% (65 out of 760 genes reported) of touch-responsive gene targets comprising calmodulins, kinases, disease resistance proteins and transcription factors supports a multitude of signaling events and highlights the importance of chromatin modifications in regulating a cascade of touch responsive transcriptional events. Furthermore, gene ontology analysis revealed similar responses (abiotic stress, defense, chemicals, wounding and other external stimuli) between the TCH:*sdg8* set of 65 genes and the complete 760 genes identified as being touch responsive. It is interesting to note that SDG8 plays a crucial role in plant defense against fungal pathogens by regulating a subset of genes within the jasmonic acid (JA) signaling pathway (Berr et al., 2010). Jasmonates have also been shown to mediate mechanostimulus-induced plant developmental responses by promoting the salient characteristics of thigmomorphogenesis in Arabidopsis, including a touch-induced delay in flowering and rosette diameter reduction (Chehab et al., 2012). Indeed our touch experiments confirm that *sdg8* mutants show perturbed leaf development, however they flower early making it difficult to infer any effect of mechanical stimulation upon flowering time in *sdg8*. A similar link was found between SDG8 mediated control of brassinosteroid regulated gene expression in Arabidopsis (Dong and Li, 2013) and 24-epibrassinolide mediates induction of TCH4/XTH22 expression (Iliev et al., 2002; Chehab et al., 2009). Overall, there is substantial evidence to implicate SDG8 mediated chromatin modifications in conferring mechano-sensing signals through an array of hormone and other signaling networks that promote thigmomorphogenesis.

The fact that the permissive chromatin-modifying enzyme SDG8 is necessary to promote full expression of such a large array of *TCH* genes partly implies that mechanical stimulation of target gene expression could be regulated by a universal regulator. Our finding that 88% of the 65 touch responsive genes regulated by SDG8 are also down-regulated, support a genuine need for chromatin modifications in the mechanical response. Plants mechanically perturbed by wind, rain, or touch induce the expression of *TCH* genes within 10–30 min post-stimulation. Our investigations showed that SDG8 was not necessary for the induction process of *TCH* gene expression; however it was clearly required for promoting maximum levels of *TCH* expression. A more comprehensive study examining a range of time points would enable identification of genes, which require SDG8 for the rapid induction of transcription rates. Chromatin modifications are well known for priming the regulatory apparatus in order to promote rapid inducible transcriptional activation of gene expression in response to environmental stresses (Kim et al., 2010; Berr et al., 2011b). The RNA polymerase would need to be docked or primed, ready to perceive a signal and rapidly activate gene expression. Nucleosome spacing would also need to be correctly configured to allow an efficient transcriptional process and these are areas worthy of future investigation. In any case, the reduced levels of *TCH* gene expression following touch stimulation in *sdg8* would affect protein levels and therefore influence downstream physiological and morphological acclimation.

Our repetitive touch experiments confirmed that several thigmomorphogenic responses were perturbed in the *sdg8* mutant. The morphological responses were varied suggesting the action of multiple signaling pathways. SDG8 mediated chromatin modifications can play a role in the acclimation of rosette development to repetitive mechanical stimulation.

Epigenetics can be viewed as a sophisticated tuner that relays environmental and developmental signals through structural adjustment of chromosomal regions so as to register, signal, or perpetuate altered activity states (Bird, 2007). Epigenetic modifications can be inherited through cell division or mitosis (e.g., chromatin modifications and DNA methylation) and in rare instances through meiosis into subsequent generations (e.g., DNA methylation) (Saze, 2008). Histone modifications can be reversible and serve to provide an additional regulatory layer to control programmed differentiation of a cell to activate or deactivate gene expression (Justin et al., 2010; Berr et al., 2011a). Our analysis of *TCH* gene expression revealed some tissue specific affects, although the expression of all three genes was significantly reduced in the shoot apex of *sdg8* mutants. The shoot apex consists of undifferentiated stem cells that can become epigenetic programmed in order to facilitate acclimation to the surrounding environment.

Given that thigomomorphogenesis is a process of cellular signal transduction and morphological acclimation it is conceivable that mechanical-induced growth responses involve some level of epigenetic memory formation. Indeed SDG8 is involved in memory forming events leading to changes in flower development (Zhao et al., 2005; Dong et al., 2008; Xu et al., 2008; Cazzonelli et al., 2009a; Grini et al., 2009; Berr et al., 2010; Tang et al., 2012; Dong and Li, 2013) and can be viewed as a combined “reader” and “writer” of the histone code possessing intrinsic H3K4 and H3K36 methyltransferase activities (Ko et al., 2010; Hoppmann et al., 2011). There is solid evidence to show that SDG8 affects both H3K4 and H3K36 tri-methylation of chromatin surrounding select gene targets and there appears to be a degree of combinatorial functions that exist between the diversity of histone methylation enzymes that exist (Kim et al., 2005; Cazzonelli et al., 2009a; Feng and Shen, 2014; Shafiq et al., 2014).

Our chromatin immunoprecipitation experiments confirmed that the *TCH3* locus requires SDG8 activity to maintain H3K4m3 marks and a permissive chromatin structure that enhances gene expression. Our bioinformatic meta-analysis of published transcriptome studies revealed that three rapidly inducible TCH gene targets (*WRKY53, STPK*, and *TCH3*) were enriched in the active marks of di- and tri-methylation of H3K4, yet lack the more repressive H3K4m1 modification. We showed that SDG8 is required to enhance *TCH* gene expression. Furthermore, the expression of genes neighboring *TCH3* were slightly reduced perhaps due to spreading of a less permissive chromatin state in the absence of *sdg8* (Kim et al., 2005; Cazzonelli et al., 2009a). While the induction of *TCH3, WRKY53* and *STPK* expression was not perturbed in the *sdg8* mutant, the level of expression following touch stimulation was clearly reduced. The fact that *TCH3* transcript levels were not so different to wild type plants following touch stimulation highlights the importance of chromatin in enhancing induced gene expression. This would imply that regulation of SDG8 could control the relative level of certain *TCH* genes following mechanical stimulation and perhaps perturb acclimation to mechanical stimulation.

Our discovery that gene expression of the potential Ca2+ sensor, CML12, first identified as TCH3 (Braam and Davis, 1990; Braam, 1992; Sistrunk et al., 1994; Antosiewicz et al., 1995) is potentially a key target of SDG8 activity is another step forward toward understanding calcium and touch mediated signaling events. *TCH3* gene expression is inducible within minutes of multiple stimuli, including touch, darkness and temperature as well as being important in mediating auxin transport and hence plant morphology. For example, TCH3 interacts with and regulates the activity of pinoid (PID), a serine/threonine protein kinase that potentially acts as a switch in regulating the activity of the PIN family of auxin regulators (Benjamins et al., 2003; Friml et al., 2004; Wisniewska et al., 2006). More recently, TCH3 was identified as having an important role in the regulation of the mechanical properties of the cell wall via an upstream signaling network dependent on Ca2+ and reactive oxygen species (ROS) (Kurusu et al., 2013). Interestingly, the expression of *TCH3* was up-regulated by overexpressors of Ca2+-permeable mechanosensitive channels (MCAs), suggesting that MCA1 stimulates the expression and activity of TCH3 through Ca2 influx (Nakagawa et al., 2007; Kurusu et al., 2012b, 2013). These channel proteins are localized in the plasma membrane and appear to be required for sensing touch, and gravity as well as osmotic shock through ROS production (Kurusu et al., 2013). Therefore regulatory proteins like MCAs and SDG8 are shedding light on how to keep genes like *TCH3* permissibly active and inducible to external stimuli. The relationship between calcium signaling, auxin transport, ROS signaling and now chromatin modifications paves the way forward to uncover the enigmatic nature behind mechanical inducible gene expression and signaling events leading to thigmomorphogenesis.

In summary, we provide new insights into the molecular nature by which a chromatin modifying enzyme SDG8, can coordinate permissive chromatin structure and enhance gene expression of mechanically responsive *TCH* genes. We reveal evidence that the active mark of H3K4m3 is required to promote the full expression of a mechanically responsive *TCH3* gene, whose protein is involved in calcium signaling events that convey the touch message. The specific role of SDG8 in thigmomorphogenesis appears to be somewhat more general when compared to carotenogenesis or flowering time, where SDG8 controls single gene loci (Cazzonelli et al., 2009a). An SDG8 mediated enrichment of H3K4 trimethylation surrounding many *TCH* gene loci could insure the rapid signaling of multiple pathways in response to a mechanical stimulus. The nature of the inducing signal(s) still remains enigmatic. How epigenetic modifications regulate transcription activation of *TCH* genes and promote memory formation in response to repetitive touch stimulation, are new areas of exciting research that will ultimately link physiological responses and morphological acclimation underlying thigmomophogenesis.

## Author contributions

Christopher I. Cazzonelli conceived idea, designed research, and wrote the paper. Christopher I. Cazzonelli, Andrea C. Roberts, and Nazia Nisar performed research, analyzed data and prepared figures. Statistical analysis by Kevin D. Murray and supervised by Justin O. Borevitz. Christopher I. Cazzonelli and Barry J. Pogson supervised Andrea C. Roberts. All authors read and commented on manuscript.

### Conflict of interest statement

The authors declare that the research was conducted in the absence of any commercial or financial relationships that could be construed as a potential conflict of interest.

## Supplementary material

Figure S1 **Experimental derived H3K4 methylation patterns surrounding selected touch inducible gene loci**. The UCSC Genome Browser (http://epigenomics.mcdb.ucla.edu/) was used to create tracts of mono-, di- and tri-methylation patterns surrounding **(A)** house keeper reference gene target SAM, **(B)** WRKY53, **(C)** STPK, and **(D)** TCH3.

Supplemental Table 1 **Bioinformatic analysis comparing genes regulated by SDG8 and responsive to mechanical stimulation**.

Supplemental Table 2 **PCR Primers used to quantify mRNA levels and chromatin modifications**.

Data Analysis S1 **Statistical analyses of morphological data using R code and nlme package**.

## Abbreviations

ccr: carotenoid and chloroplast regulation
sdg8: set domain group eight
efs: early flowering under short days
ashh2: ASH homolog2
H3K4: histone-3 lysine-4 methylation
H3K36: histone-3 lysine-36 methylation
H3K9: histone-3 lysine-9 methylation
H3K27: histone-3 lysine-27 methylation
HKM: histone lysine methylation
HKMTs: histone lysine methytransferases
SET: Su(var)3-9, E(Z), trithorax
DAG: days after germination.
