# Supplementary material for: A chromatin modifying enzyme, SDG8, is involved in morphological, gene expression, and epigenetic responses to mechanical stimulation
Source: Front Plant Sci. 2014 Oct 21;5:533. doi: 10.3389/fpls.2014.00533 (PMC4204441; doi:10.3389/fpls.2014.00533)
Supplement: Supplemental Table 1 — Bioinformatic analysis comparing genes regulated by SDG8 and responsive to mechanical stimulation. [file DataSheet2.DOC]

**Supplemental Table 2**. PCR Primers used for quantitative real time PCR of gene expression and chromatin immunoprecipitation

| **Target** | **Target Name** | **Accession** | **Sequence (5'-->3')** | **Direction** | **Amplicon** |
| --- | --- | --- | --- | --- | --- |
|  |  | **Number** |  |  | **Size (bp)** |
| **qRT-PCR (House Keeper Genes)** | | | | | |
| **Cyclo** | Cyclophilin | At2g29960 | TCTTCCTCTTCGGAGCCATA | Forward | 250 |
|  |  |  | AAGCTGGGAATGATTCGATG | Reverse |  |
| **PP2A** | Protein Phosphatase | At1g13320 | CTTCGTGCAGTATCGCTTCTC | Reverse | 249 |
|  |  |  | ATTGGAGAGCTTGATTTGCG | Forward |  |
| **TIP41** | unknown | AT4g34270 | CAACGCCATACTGTGGAAGTG | Forward | 260 |
|  |  |  | AAATCGCAAGAGGAGGAACC | Reverse |  |
| **qRT-PCR (Mechanical Inducible Genes)** | | | | | |
| **WRKY** | DNA Binding Protein | AT4G23810 | TTGCCGATGGAGGAGGTTCTA | Forward | 258 |
|  |  |  | TCTGGACTTGTTTCGTTGCCC | Reverse |  |
| **STPK** | Protein Kinase | AT2G47060 | CCCGAGTTCTTGGGACTTTTG | Forward | 244 |
|  |  |  | TTTGGAGGGTAATCGCCACC | Reverse |  |
| **TCH3** | Calmodulin Like Protein | AT2G41100 | TACCGTGATGTTTTCCCTCG | Forward | 268 |
|  |  |  | GAGCTCATTCACGGTAATGTAACC | Reverse |  |
| **CHiP (Mechanical Inducible Genes)** | | | | | |
| **TCH3-Prom** | Touch Inducible 3 | AT2G41100 | TTAGGCAAATGTCCACTCACCC | Forward | 270 |
| **TCH3-Prom** |  |  | ACGAACCAGGACTTGAAGCC | Reverse |  |
| **TCH3-Exon1** | Touch Inducible 3 | AT2G41100 | GTGATGTTTTCCCTCGGTAA | Forward | 269 |
| **TCH3-Exon1** |  |  | TTAGCGAGAAAGGGACGATG | Reverse |  |
| **TCH3-Exon4** | Touch Inducible 3 | AT2G41100 | GGTGAAACCCAAACAAAAGC | Forward | 260 |
| **TCH3-Exon4** |  |  | GGCGAAGCGATGATATTG | Reverse |  |
| **CHiP (House Keeper Genes)** | | | | | |
| **SAM** | S-Adenosyl Methionine | At4g01850 | TGAAGTCCAAAAGCAAAAACC | Forward | 197 |
|  |  |  | GACGGAGAAGAAGAGCGAAA | Reverse |  |
| **CRTISO-Prom** | Carotenoid Isomerase | AT1g06820 | ACAAAGATGCGGTCTCAACG | Forward | 216 |
|  |  |  | CCCAACCAAGTTGGAATAGCT | Reverse |  |
