# Supplementary material for: A chromatin modifying enzyme, SDG8, is involved in morphological, gene expression, and epigenetic responses to mechanical stimulation
Source: Front Plant Sci. 2014 Oct 21;5:533. doi: 10.3389/fpls.2014.00533 (PMC4204441; doi:10.3389/fpls.2014.00533)
Supplement: Supplemental Table 2 — PCR Primers used to quantify mRNA levels and chromatin modifications. [file DataSheet3.ZIP › touch_paper_stats.pdf]

Cazzonelli et al 2014 Figure 1

The model fitted should be a two-factor linear mixed effect model (Genotype * treatment are the two factors, w/ interaction), with a random effect term for the tray (batch) effect. This removes the error in each of the two model factors caused by tray effect whilst fitting the model.

So, lets get the data:

```
fig1_data <- read.csv("TouchData.csv")
fig1_data$Genotype <- relevel(fig1_data$Genotype, ref="WT")
fig1_data$Block <- as.numeric(fig1_data$Genotype)
fig1_data$Block12 <- as.numeric(fig1_data$Genotype) * as.numeric(fig1_data$Tray)
summary(fig1_data)
```

```
##    Genotype        Treatment        Tray            Rep
##  WT    :252   NotTouched:215   Min.   :1.00   Min.   : 1.0
##  ccr1.1:197   Touched   :234   1st Qu.:2.00   1st Qu.:10.0
##                                Median :4.00   Median :19.0
##                                Mean   :3.58   Mean   :19.6
##                                3rd Qu.:5.00   3rd Qu.:29.0
##                                Max.   :6.00   Max.   :42.0
##         Measurement      Value           Block          Block12
##  LeafLength   :150   Min.   : 4.05   Min.   :1.00   Min.   : 1.0
##  LeafWidth    :149   1st Qu.:10.35   1st Qu.:1.00   1st Qu.: 3.0
##  PetioleLength:150   Median :12.58   Median :1.00   Median : 5.0
##                      Mean   :14.10   Mean   :1.44   Mean   : 5.2
##                      3rd Qu.:18.33   3rd Qu.:2.00   3rd Qu.: 6.0
##                      Max.   :27.17   Max.   :2.00   Max.   :12.0
```

```
table(paste(fig1_data$Genotype, fig1_data$Treatment))
```

```
##
## ccr1.1 NotTouched    ccr1.1 Touched     WT NotTouched        WT Touched
##                89               108               126               126
```

```
# this is your N per treatment
table(paste(fig1_data$Block, fig1_data$Tray, fig1_data$Measurement))
```

```
##
##    1 1 LeafLength     1 1 LeafWidth 1 1 PetioleLength    1 2 LeafLength
##                15                15                15                13
##     1 2 LeafWidth 1 2 PetioleLength    1 3 LeafLength     1 3 LeafWidth
##                13                13                14                14
## 1 3 PetioleLength    1 4 LeafLength     1 4 LeafWidth 1 4 PetioleLength
##                14                14                14                14
##    1 5 LeafLength     1 5 LeafWidth 1 5 PetioleLength    1 6 LeafLength
##                14                14                14                14
##     1 6 LeafWidth 1 6 PetioleLength    2 1 LeafLength     2 1 LeafWidth
##                14                14                 8                 8
## 2 1 PetioleLength    2 2 LeafLength     2 2 LeafWidth 2 2 PetioleLength
##                 8                 9                 8                 9
##    2 3 LeafLength     2 3 LeafWidth 2 3 PetioleLength    2 4 LeafLength
```

```
##                 13                 13                 13                 14
##     2 4 LeafWidth 2 4 PetioleLength    2 5 LeafLength     2 5 LeafWidth
##                 14                 14                 10                 10
## 2 5 PetioleLength    2 6 LeafLength     2 6 LeafWidth 2 6 PetioleLength
##                 10                 12                 12                 12
```

```
min(table(paste(fig1_data$Block, fig1_data$Tray, fig1_data$Measurement)))
```

```
## [1] 8
```

```
max(table(paste(fig1_data$Block, fig1_data$Tray, fig1_data$Measurement)))
```

```
## [1] 15
```

Now, for each phenotype, we want to fit the linear mixed effects model. We'll need the package `nlme`, which contains the `lme` function we use below.

```
library(nlme)

phenotypes <- unique(as.character(fig1_data$Measurement))
for (pheno in phenotypes) {
  pdata <- fig1_data[fig1_data$Measurement == pheno,]
  fit <- lme(Value ~ Genotype * Treatment, data = pdata, random =~ 1|Tray)
  print(pheno)
  print(summary(fit))
}
```

```
## [1] "LeafLength"
## Linear mixed-effects model fit by REML
##  Data: pdata
##      AIC   BIC logLik
##    813.2 831.1 -400.6
##
## Random effects:
##  Formula: ~1 | Tray
##         (Intercept) Residual
## StdDev:      0.6659    3.549
##
## Fixed effects: Value ~ Genotype * Treatment
##                                 Value Std.Error  DF t-value p-value
## (Intercept)                    22.319    0.6693 142   33.35  0.0000
## Genotypeccr1.1                 -6.191    0.8509 142   -7.28  0.0000
## TreatmentTouched               -1.319    0.9464   4   -1.39  0.2359
## Genotypeccr1.1:TreatmentTouched  3.481    1.1727 142    2.97  0.0035
##  Correlation:
##                                (Intr) Gnt1.1 TrtmnT
## Genotypeccr1.1                 -0.527
## TreatmentTouched               -0.707  0.373
## Genotypeccr1.1:TreatmentTouched  0.383 -0.726 -0.541
##
## Standardized Within-Group Residuals:
##      Min       Q1      Med       Q3      Max
```

```
## -3.25228 -0.53027  0.06779  0.72290  2.00834
##
## Number of Observations: 150
## Number of Groups: 6
## [1] "PetioleLength"
## Linear mixed-effects model fit by REML
##  Data: pdata
##   AIC   BIC logLik
##   663 680.9 -325.5
##
## Random effects:
##  Formula: ~1 | Tray
##         (Intercept) Residual
## StdDev:      0.8795    2.091
##
## Fixed effects: Value ~ Genotype * Treatment
##                                 Value Std.Error  DF t-value p-value
## (Intercept)                    15.109    0.6018 142  25.106  0.0000
## Genotypeccr1.1                 -3.706    0.5026 142  -7.375  0.0000
## TreatmentTouched               -4.579    0.8510   4  -5.381  0.0058
## Genotypeccr1.1:TreatmentTouched  1.963    0.6922 142   2.836  0.0052
##  Correlation:
##                                (Intr) Gnt1.1 TrtmnT
## Genotypeccr1.1                 -0.345
## TreatmentTouched               -0.707  0.244
## Genotypeccr1.1:TreatmentTouched  0.251 -0.726 -0.354
##
## Standardized Within-Group Residuals:
##      Min       Q1      Med       Q3      Max
## -2.98953 -0.50926  0.02263  0.70973  2.40843
##
## Number of Observations: 150
## Number of Groups: 6
## [1] "LeafWidth"
## Linear mixed-effects model fit by REML
##  Data: pdata
##     AIC   BIC logLik
##   556.5 574.3 -272.2
##
## Random effects:
##  Formula: ~1 | Tray
##         (Intercept) Residual
## StdDev:   5.696e-05    1.505
##
## Fixed effects: Value ~ Genotype * Treatment
##                                 Value Std.Error  DF t-value p-value
## (Intercept)                    12.731    0.2322 141   54.82  0.0000
## Genotypeccr1.1                 -3.383    0.3633 141   -9.31  0.0000
## TreatmentTouched               -1.366    0.3284   4   -4.16  0.0141
## Genotypeccr1.1:TreatmentTouched  1.801    0.4989 141    3.61  0.0004
##  Correlation:
##                                (Intr) Gnt1.1 TrtmnT
## Genotypeccr1.1                 -0.639
## TreatmentTouched               -0.707  0.452
```

```
## Genotypeccr1.1:TreatmentTouched  0.465 -0.728 -0.658
##
## Standardized Within-Group Residuals:
##     Min      Q1     Med      Q3     Max
## -2.9119 -0.5388  0.1646  0.7409  2.0657
##
## Number of Observations: 149
## Number of Groups: 6
```

So, we have the following "ANOVA" table (though this is NOT an anova). The values are the size of the effect (in mm) and the p-value assoicated with the effect. This is all relative to non-touched wild-type plants.

Phenotype	Genotype	Treatment	Genotype:Treatment
Leaf Len	-6.2 (0.00)	-1.3 (0.24)	3.5 (0.00)
Leaf Width	-3.4 (0.00)	-1.4 (0.01)	1.8 (0.00)
Petiole Len	-3.7 (0.00)	-4.6 (0.01)	2.0 (0.01)

What does this mean?

This fits the model "Phenotype is explained by Genotype + Treatement + an interaction between the two + a random effect from the tray". So, in the above table, the effects (relative to WT Non-touched) indicate a model estimation of roughly how large the effect of genotype, treatment, and the interaction is the size and direction of the interaction. In this case, for Leaf Len, both genotype and treatment have negative effects (SDG8 reduces leaf length, as does touching). Therefore, SDG8 touched plants have longer (more positive) leaves than just the addition of the effects of genotype and treatment.

Plots

OK, now some plots. These are NOT actual values, they are fitted values, based off the same model we used above. This means that these values don't nicely correspond to any exact value in a table of raw data, but means that they are more accurately annotated with stats from the model above.

```
for (pheno in phenotypes) {
  pdata <- fig1_data[fig1_data$Measurement == pheno,]
  N = nrow(pdata)
  fit <- lme(Value ~ Treatment * Genotype, data = pdata, random =~ 1|Tray)
  fit.data <- tapply(predict(fit),
                     paste(pdata$Genotype, pdata$Treatment, sep='\n'),
                     mean)
  # re-order the factors so it's WT then ccr2
  fit.data <- fit.data[c(3,4,1,2)]
  names(fit.data) <- c("WT\nCtrl", "WT\nTch", "sdg8\nCtrl", "sdg8\nTch")
  # plot to screen
  xx <-  barplot(fit.data, ylim = c(0,25), beside = T,
                col=c("#000000", "#FFFFFF", "#000000", "#FFFFFF"))
  se = fit$sigma / sqrt(N)
  segments(xx, fit.data, xx, fit.data + (2 * se))
```

```
  segments(xx, fit.data, xx, fit.data - (2 * se))
  title(paste("LME-predicted values for", pheno))
  # re-plot to pdf
  pdf(paste0(pheno, '_fitted.pdf'), width=3.5)
  xx <-  barplot(fit.data, ylim = c(0,25), beside = T,
                col=c("#000000", "#FFFFFF", "#000000", "#FFFFFF"))
  se = fit$sigma / sqrt(N)
  segments(xx, fit.data, xx, fit.data + (2 * se))
  segments(xx, fit.data, xx, fit.data - (2 * se))
  title(paste(pheno))
  dev.off()
  #
  print(paste("Predicted values for", pheno))
}
```

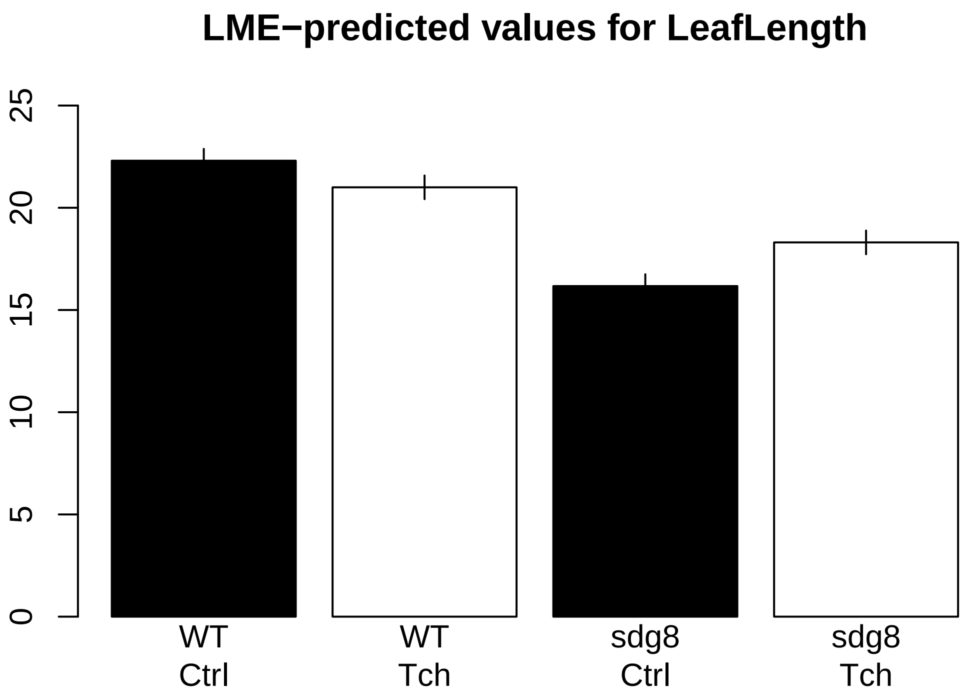


```
## [1] "Predicted values for LeafLength"
```

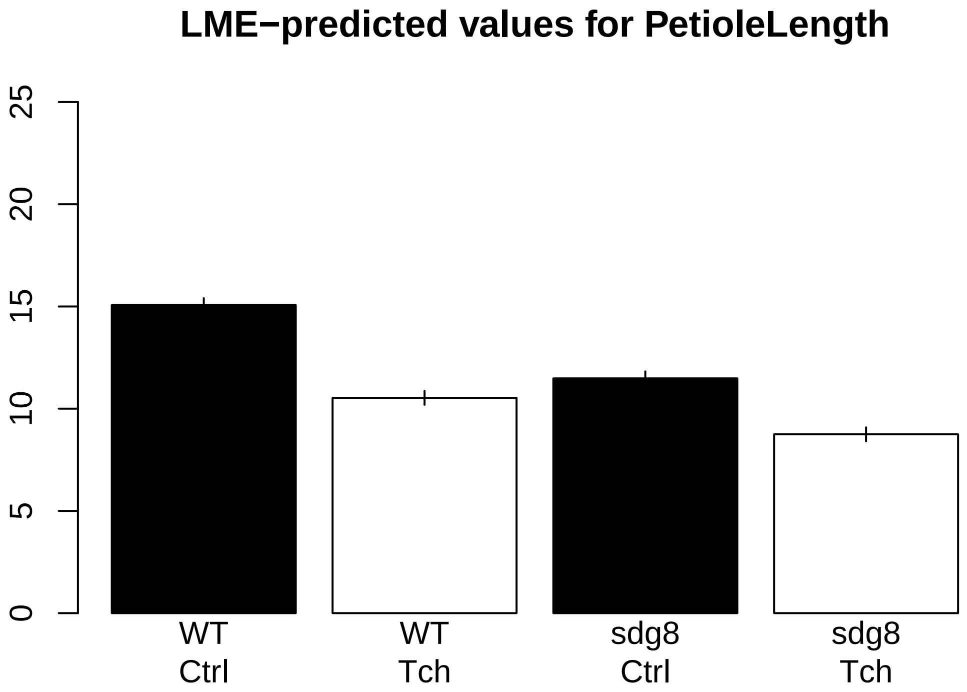


```
## [1] "Predicted values for PetioleLength"
```

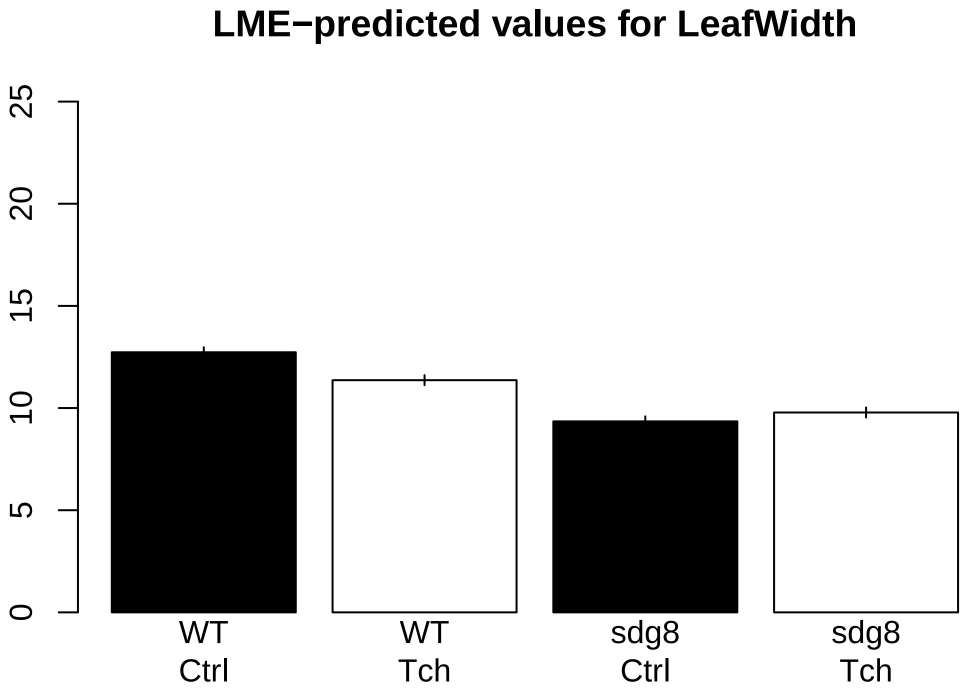


```
## [1] "Predicted values for LeafWidth"
```
