# Supplementary figures and images for: A chromatin modifying enzyme, SDG8, is involved in morphological, gene expression, and epigenetic responses to mechanical stimulation
Source: Front Plant Sci. 2014 Oct 21;5:533. doi: 10.3389/fpls.2014.00533 (PMC4204441; doi:10.3389/fpls.2014.00533)

# LeafLength

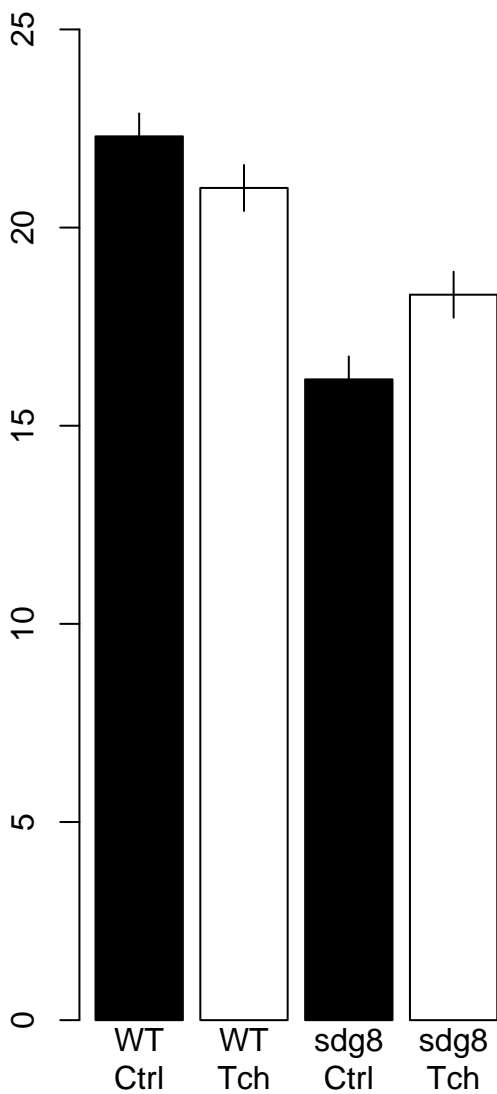

Supplement: Supplemental Table 2 — PCR Primers used to quantify mRNA levels and chromatin modifications. [file DataSheet3.ZIP › LeafLength_fitted.pdf]

## LeafWidth

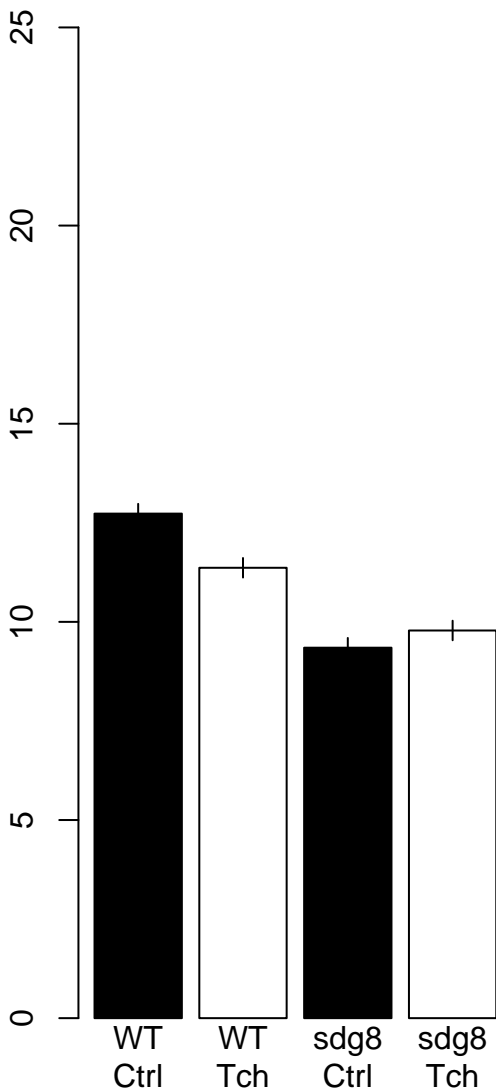

Supplement: Supplemental Table 2 — PCR Primers used to quantify mRNA levels and chromatin modifications. [file DataSheet3.ZIP › LeafWidth_fitted.pdf]

# PetioleLength

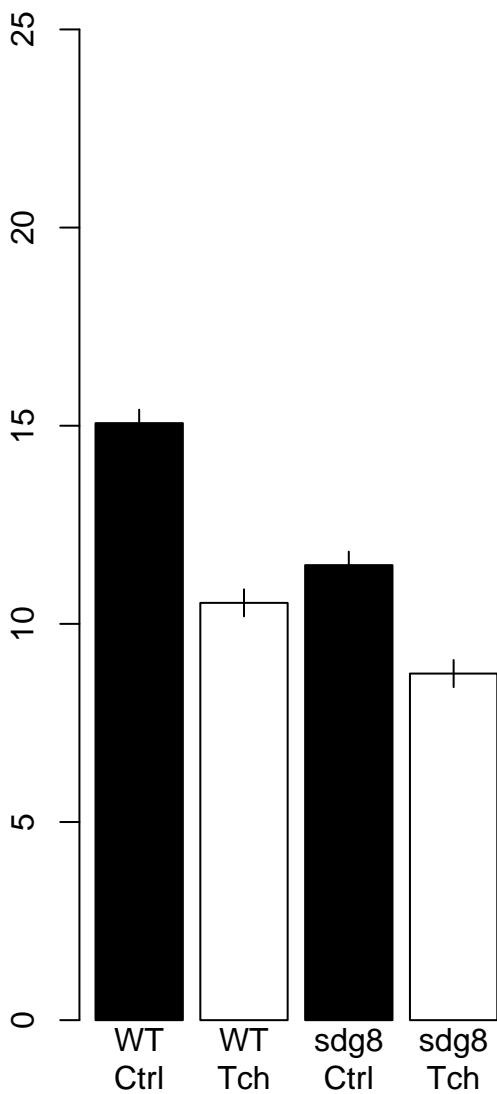

Supplement: Supplemental Table 2 — PCR Primers used to quantify mRNA levels and chromatin modifications. [file DataSheet3.ZIP › PetioleLength_fitted.pdf]

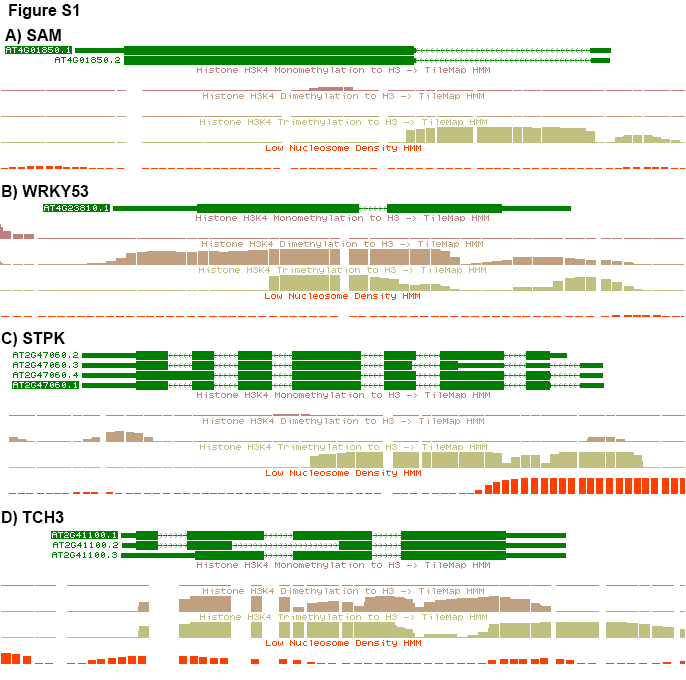

Supplement: Data Analysis S1 — Statistical analyses of morphological data using R code and nlme package. [file Image1.JPEG]
